# On the continuity of rehabilitation and meeting the patients’ needs: Online psychosocial treatment during the COVID-19 outbreak

**DOI:** 10.1192/j.eurpsy.2021.1800

**Published:** 2021-08-13

**Authors:** A. Palin, N. Shilko, A. Pozharskaya, L. Burygina, N. Semenova

**Affiliations:** 1 Psychotherapy Center, State Budgetary Health Organization Psychiatric Clinical Hospital №4 named after P.B. Gannushkin, Moscow, Russian Federation; 2 Administration, State Budgetary Health Organization Psychiatric Clinical Hospital №4 named after P.B. Gannushkin, Moscow, Russian Federation; 3 Psychiatry, Moscow Research Institute of Psychiatry – a branch of V.Serbsky National Medical Research Centre for Psychiatry and Narcology, Moscow, Russia, Moscow, Russian Federation

**Keywords:** COVID-19, Rehabilitation programs, Patients’ needs, Online psychosocial treatment

## Abstract

**Introduction:**

The COVID-19 pandemic has challenged our model of face-to-face psychosocial treatment and rehabilitation format. To adapt to the current situation, as professionals, we have decided to transform the format into a virtual one that will offer the continuity of rehabilitation and therapy. Two clinical psychologists held online sessions and a special chat created in the IM messenger where patients could safely interact with each other.

**Objectives:**

This pilot study aimed to evaluate the effect of online sessions in a sample of outpatients engaged in rehabilitation programs.

**Methods:**

Data from 50 patients (F20-F25, aged from 25 to 45) treated with a new online psychosocial program, including i. psychoeducation, ii. learning skills of the behavior under the circumstances of isolation, iii. training skills of effective communication and emotional regulation, and assessed for depression, anxiety, hopelessness, hostility (BDI, STAI, BHS, BDHI), and self-esteem, were analyzed for this study. Motivational enhancement techniques were also used to engage the patients in this new treatment format.

**Results:**

According to the preliminary data, we point out a statistically meaningful reduction in depression (p=0,003), anxiety (p=0,001), and hostility (р=0,001); self-esteem, evaluated with the Dembo-Rubinstein method, was improved (p=0,002); the T Wilcoxon criterion used for rating the magnitude.

**Conclusions:**

Our results indicate that establishing a new online psychosocial program over the last few months positions us to respond effectively to such a new challenge and suggest that rehabilitative programs targeting patients’ needs may continue in this time of uncertainty.

**Disclosure:**

No significant relationships.

